# The outcome of kidney transplant from living donors with pelvi-ureteric junction dysfunction

**DOI:** 10.1007/s11255-020-02522-x

**Published:** 2020-06-10

**Authors:** Miroslav Tisljar, Hatem Ali, Charlie Gledhill-Flynn, Mila Garreus, Arvind Ponnusamy, Aimun Ahmed

**Affiliations:** 1 Renal Department, Royal Preston Hospital, Lancashire Teaching Hospitals NHS Foundation TrustSharoe Green, Fulwood, Preston, PR2 9HT UK; 2 Nephrology Department, University Hospital Dubrava, Zagreb, Croatia; 3 Nephrology Department, Faculty of Medicine, Ain Shams University, Cairo, Egypt; 4 Manchester Medical School, University of Manchester, Manchester, UK; 5 Institute of Cardiovascular Science, University of Manchester, Manchester, UK

**Keywords:** Kidney transplant, Living donors, Pelvi-ureteric junction dysfunction, Kidney function

## Abstract

**Purpose:**

To assess the effect of receiving a kidney with PUJ dysfunction on the recipient renal graft function.

**Methodology:**

198 patients, who underwent renal transplantation from 1st January 2004 to 31st December 2014 in a single Center in the North West of England, were retrospectively reviewed using a computerized database. Split kidney function and the PUJ dysfunction for the donors were assessed using Tc-99 m MAG3 renogram. Each recipient with PUJ dysfunction was matched with a control recipient by age, gender, and number of days after transplantation. Both groups were followed up for 3.5 years post-transplantation.

**Results:**

Of the 198 recipients included in the study, 19 recipients received kidneys from donors with PUJ dysfunction. Prevalence of PUJ dysfunction was 9.5% and it was more common in males than females. There was no difference between the case group and the control group in terms of age, gender, and follow-up time post-transplantation. There was also no difference between the case group and the control group in mean creatinine (130 µmol/l and 138 µmol/l respectively, *p* = 0.305) or the mean eGFR (48.6 ml/min and 47.5 ml/min respectively, *p* = 0.054) at 3.5 year post-kidney transplantation.

**Conclusion:**

This study showed that PUJ dysfunction of renal allograft has a negligible effect on graft function over 3.5 years period post-transplantation. A prospective randomized trial is needed to test these findings. In the presence of widened gap between demand and supply in renal transplantation, PUJ dysfunction in potential donors should not preclude them from donation.

## Introduction

Kidney transplantation is the preferred option for patients with end-stage renal disease. It provides better quality of life and better survival when compared to dialysis [1, 2]. Patients with end-stage renal disease are with compelling comorbidities and it is vital for potential recipients to be properly evaluated to assess and manage these comorbidities and risk factors that can affect the outcome of renal transplant [3, 4]. However, there is a wide gap between number of patients with end-stage renal disease seeking kidney transplant and the number of potential kidney donors, and as this gap continues to widen, it has lead to accepting expanded criteria donors and marginal donors [5]. On the other hand, nephrectomy for living kidney donors is not free of risks as it exposes them to surgical hazards and potential long-term effect on renal functions and all-cause mortality, wherefore thorough assessment for kidney donor is a paramount [6].

Pelvi-ureteric junction (PUJ) dysfunction is characterized by disruption of the flow of the urine through partial or complete blockage at the site of ureteropelvic connection [7, 8]. It occurs in 1in 500 live births and it is more common in males then females [8]. The diagnosis of partial PUJ dysfunction is still challenging as the optimal test to assess upper urinary tract dysfunction remains not existing. Although renogram tests are the commonest method in assessing upper urinary tract obstruction, ambivalent results can occur in 10–17% of cases [10]. Technetium-99 m mercaptoacetyltriglycine (Tc-99 m MAG3) is one of the popular tests used in assessing split kidney functions in potential kidney donors [11]. It also can detect anatomical abnormalities in living kidney donors. A few studies and case reports demonstrated the effect of donor kidney PUJ dysfunction on recipient post-transplant kidney function [10, 12, 13]. The aim of the study is to assess the effect of receiving a kidney with PUJ dysfunction.

## Methodology

Renal function of 198 patients who underwent live donor renal transplantation from 1st January 2004 to 31st December 2014 in a single center in the North West of England was retrospectively reviewed using a computerized database. For kidney recipients, the following measures were collected: age at transplantation, gender, and number of days since the transplant.

For the donors, data about split kidney functions and presence of anatomical abnormalities were collected. Anatomical abnormalities, blood supply for the retrieved kidneys, and split kidney functions for the donors were assessed using abdominal ultrasound, CT renal arteries’ angiogram, and Tc-99 m MAG3 renogram. Recipients from live kidney donors with PUJ dysfunction were included, while those with other ureteropelvic anatomical abnormalities detected on renal ultrasound, CT angiography, or MAG3 renogram were excluded from the study. Each live donor recipient of graft with PUJ dysfunction was matched with a control recipient (live donor recipient without PUJ) by age, gender, and number of days since the transplant. The degree of renal function for both groups was assessed by serum creatinine levels and estimated glomerular filtration rate (eGFR) using four variable MDRD equation. Both groups were followed up for average 3.5 years post-transplant. Serum creatinine was reviewed at 3 monthly intervals during follow-up period whereby mean creatinine and eGFR were calculated (baseline and last values of both groups were included in overall average values). Graft loss was defined by patient death with functioning graft or commencement of dialysis dependency. Statistical analysis of the data was completed using an unpaired *T* test to show the difference between the two independent samples both of which followed a normal distribution. A Mann–Whitney *U* test was also used to check for significance for non-parametric data. *p* value < 0,05 was considered as statistically significant.

## Results

Of the 198 kidney recipients included in the study, 19 recipients received kidneys from donors with anatomical abnormalities that appeared on the MAG3 renogram. MAG3 abnormalities assessed in this study included PUJ dysfunction in form of fullness of pelvi-caliceal system, initial tracer accumulation, slow or sluggish excretion, and minor/small/minimal impairment. Prevalence of PUJ dysfunction was 9.5% and it was more common in males. Comparison between characteristics of both groups is shown in Table 1.

**Table 1:** Comparison between case and control groups

	PUJ dysfunction	Matched control group
Total number	19	19
Average age (years)	53.1 (+/− 3.61)	54.8 (+/− 3.44)
Male	11	11
Female	8	8
Average follow-up time since transplantation (days)	1173.5 (+/− 100.37)	1266 (+/− 104.03)

*PUJ* pelvi-ureteric junction

After 3.5 years of follow-up, in the case group, mean creatinine value was 130 µmol/l and mean eGFR was 48.6 ml/min, while in the control group, mean creatinine value was 138 µmol/l and mean eGFR was 47.5 ml/min, respectively (Table 2).

**Table 2:** Comparison of mean creatinine and mean eGFR value in kidney recipients from living donors with PUJ dysfunction and control groups after 3.5 year follow-up

	Recipients from donors with PUJ dysfunction	Recipients from donors without PUJ dysfunction	95% confidence interval	*p*
Mean serum creatinine	130 µmol/L	138 µmol/L	$-157.23 \mathrm{t}\mathrm{o} 50.93$	0.305
Mean eGFR	48.6 ml/min	47.5 ml/min	$-15.12 \mathrm{t}\mathrm{o} 17.37$	0.054

*PUJ *pelvi-ureteric junction, *eGFR *estimated glomerular filtration rate

## Discussion

The precise diagnosis of PUJ dysfunction is not always straightforward due its rare prevalence and lack of the existence an ideal diagnostic test. PUJ dysfunction is found more on the left than on the right side and the percentage of bilateral involvement ranges from 10 to 40% [14]. Most of the cases of PUJ dysfunction are secondary to congenital abnormalities (the most common cause of antenatal hydronephrosis) leading to internal narrowing of the ureter at the pelvi-ureteric junction and rarely can be caused by external compression of surrounding vessels or lymph nodes [8]. Kidney function is deteriorated mostly in cases of complete urine flow obstruction, but even the cases secondary to partial obstruction can also cause slow derangement in kidney functions. However, sometimes, a state of equilibrium can occur leading to stability in kidney functions [7]. The occurrence of equilibrium state relies on rate of urine output, stage of PUJ dysfunction, and the conformity of the renal pelvis [7].

Measurement of divided renal function is advised in instances where there is a considerable difference in size between the two kidneys or an obvious anatomical abnormality has been noted on ultrasound [15].

**Fig. 1:**
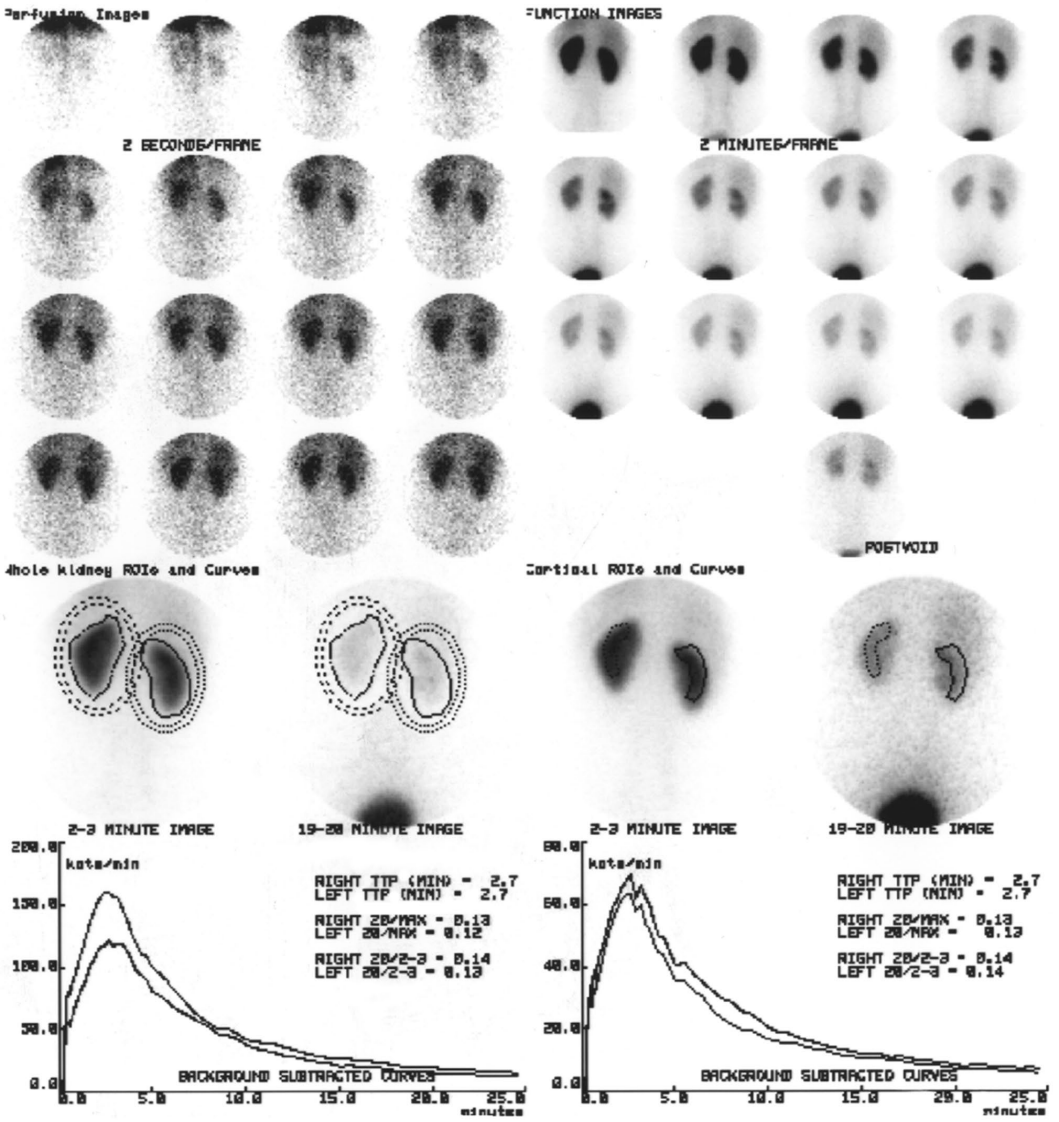
MAG3 renogram in an individual with normal kidneys. As well as showing the progression of tracer through the kidneys, we also see the renogram curves for the cortex and the whole kidney on the bottom of the image; in this individual, these curves are essentially the same showing a normal scan[17]

**Fig. 2:**
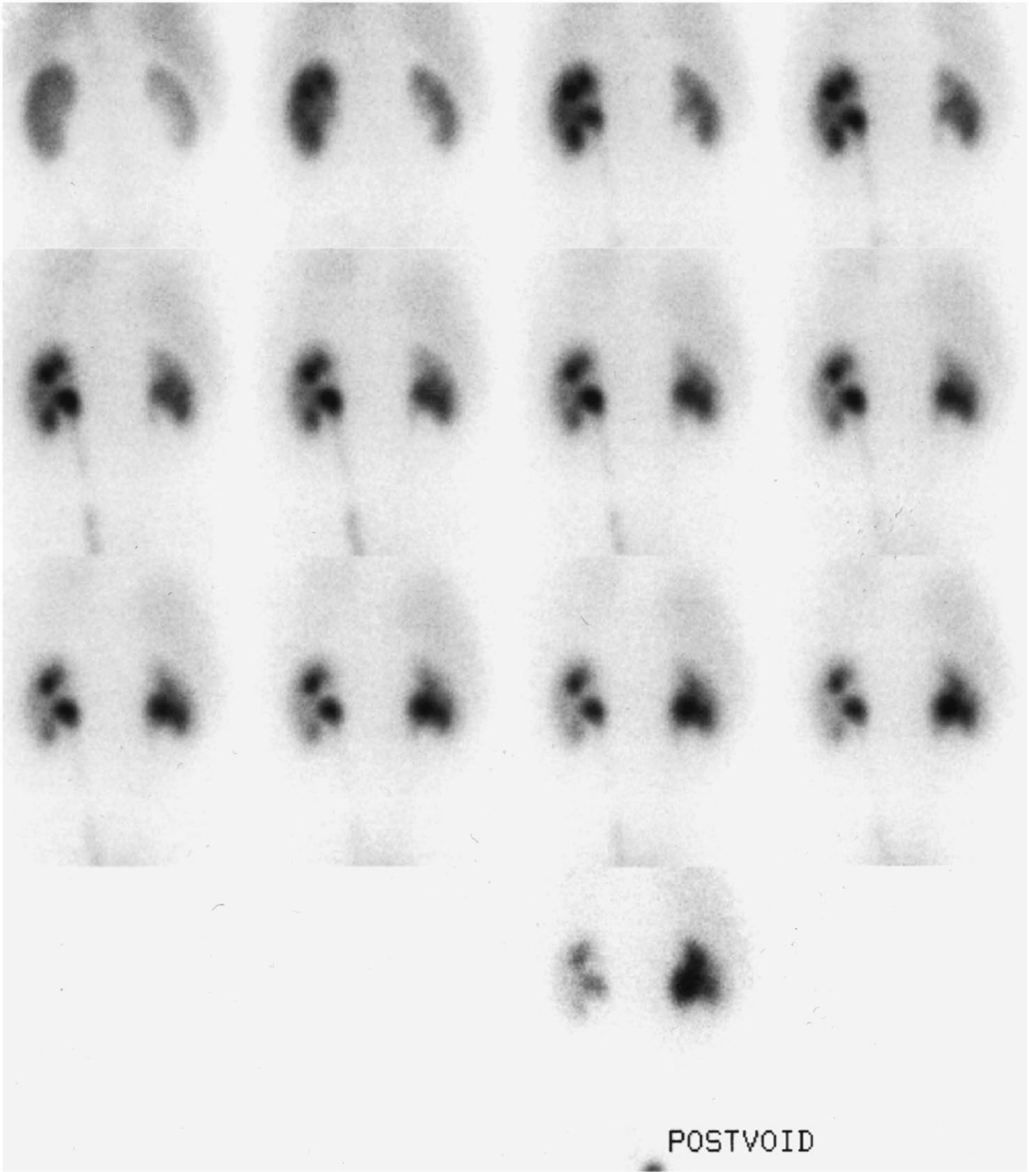
‘Sequential 2-min images show prompt uptake in both kidneys with marked retention in the right renal pelvis.’ This sequences of images from a MAG3 scan show one of the possible definitions of pyelo-ureteric junction dysfunction [17]

The diuretic renogram is the cornerstone test to determine upper urinary tract obstruction. However, equivocal results are found in 15–17% of patients [10]. In the presence of PUJ dysfunction or partial PUJ obstruction, split kidney functions should be considered when assessing the kidney to be retrieved. Split kidney function can be measured by conjoining a 51Cr-EDTA GFR measurement with a 99mTc-DMSA scan [16] or which is even more common (also done in our case series) by Tc-99 m MAG3 [11]. An illustration how looks like normal MAG3 renogram and an example of MAG3 renogram of the kidney with PUJ are depicted in Figs. 1 and 2 [17]. In general population, asymptomatic patients require a baseline MAG3 scan and serial monitoring with ultrasound scanning. Urological intervention is needed if there is increasing hydronephrosis with an anterior posterior diameter (APD) > 3 cm, function below 40% or a drop in function of > 10% on repeat MAG3 [18]. When considering pyelo-ureteric junction dysfunction and MAG3 abnormalities in terms of living kidney donors, the affected kidney should be taken for transplant. This decision is based on leaving the donor with the greatest residual kidney function as possible. When kidney function is normal and there is a serious disparity in glomerular filtration rate between both kidneys more than 10%, it is advisable to retrieve the kidney with the lower glomerular filtration rate [15]. In our center, the donor kidney with the lowest split function is taken for transplant, regardless of the degree of difference between the two kidneys, with the stipulation that a kidney with a function of less than 40% is contraindicated for transplant.

In our study, the incidence of PUJ dysfunction was 9.5%. The average age of recipients was 53 years with slightly male predominance and they all were on regular kidney transplantation waiting list. All patients in both groups were low immunological risk, and they were all first transplants. All the 19 recipients with PUJ dysfunction renal allografts were on dual therapy of tacrolimus and mycophenolate mofetil; none were on steroids as maintenance (steroids were withdrawn early within the first few weeks). In the control group, dual therapy was matched with the study group. None of the study group or the control patient had delayed graft function neither experienced acute rejection or underwent renal allograft biopsy from some other reason. Likewise, during an early post-transplant period, none of our patients have developed any adverse urological complications.

On review of post-transplant MAG3 scans in recipients of a kidney with PUJ, nine kidneys had normal scans showing good function and two kidneys still had some form of abnormality. The abnormalities found were not related to the documented pre-transplant abnormalities. Data were unavailable for eight kidneys. It is quite possible that the abnormalities found on the MAG3 scan are corrected during the surgery, and so, the previously dysfunctional kidneys would then be directly comparable to kidneys used for transplant with no pyelo-ureteric junction dysfunction. On average of 1173.5 days of follow-up, mean serum creatinine value was 130 µmol/l with mean eGFR 48.6 ml/min what was comparable to control study group who had mean serum creatinine level 138 µmol /l, *p* = 0,305; with mean eGFR 47.5 ml/min, *p* = 0.054.

Literature about PUJ dysfunction in live donor kidney transplantation is limited. In the transplanted kidney, PUJ dysfunction can occur post-transplantation secondary to denervation of the graft [10]. This leads in autonomic dysfunction with deranged contractility that triggers urine flow obstruction what can result with serious complications in the recipient.

However, PUJ dysfunction and/or obstruction can be corrected during transplantation or after transplantation with good outcome. Shabtai et al. reported a case where a deterioration of kidney function in a renal transplant recipient occurred 6 months post-transplant and this was secondary to PUJ obstruction [10]. Investigations done to the donor prior to graft retrieval showed the presence of an asymptomatic renal pelvis dilatation. Surgical treatment consisted of a Foley nondismembered Y-V pyeloplasty followed by improvement of recipient kidney function. In one another similar case report in which the donors’ intravenous urogram (IVU) showed dilatation of the renal pelvis, the PUJ was dismembered and a pyelo-native ureterostomy was performed over a stent [12]. Postoperative period was uneventful. Biopsy of the excised segment showed mild suburothelial chronic inflammation with no evidence of viral cytopathic changes. In both papers it was hypothesized that the causes of renal function deterioration could be secondary to renal denervation of the graft, postoperative periureteral fibrosis or secondary to any immune reaction or natural progression of the partial obstruction. Ho et al. reported the use of a kidney with diagnosed PUJ dysfunction which was used for transplant [13]. During the transplantation surgery, Anderson Hynes pyelo-native ureterostomy was performed with good postoperative success. Soukup et al. presented very interesting case report with male patient who had recurrent episodes of pyelonephritides and stone formation due to unilateral hydronephrosis (PUJ dysfunction was detected) and total nephrectomy was the therapy of choice [19]. Following preoperative discussion, both donor and kidney recipient (69-year-old woman) consented to transplantation. During surgery, PUJ stenosis was excised and a direct pelvi-vesical anastomosis was formed. Both patients made uncomplicated recoveries, the kidney donor was symptom free and was delighted with the successful transplant outcome. There are reported case series with kidney recipients who received kidneys (living or deceased donors) without anatomical abnormalities but developed PUJ dysfunction after surgery was done. The PUJ dysfunction was resolved by the balloon dilatation [20] or endopyelotomy with good long lasting results [21].

In our 19 kidney recipients’ cohort over the 3.5 years of follow-up period, none of the recipients needed any of urological intervention. None of live donor recipients with PUJ dysfunction transplant have been developed ESRD or died with functioning graft. Moreover, we did not found any case of overt stone formation or serious urinary tract infection which would have required hospitalization.

Limitations of our study include its retrospective design, relatively short follow-up period, and the relatively small sample size which could be attributed to the scarcity of this abnormality. However, according to our best knowledge, this is so far the biggest case series report in kidney transplants from living donors with PUJ dysfunction.

In conclusion, our study showed that PUJ dysfunction in a renal allograft has a negligible effect on graft function over 3.5 year period post-transplantation. More prospective randomized long-term trials are needed to test these findings. In the presence of widened gap between demand and supply in renal transplantation, PUJ dysfunction in potential living kidney donors should not preclude them from donation.
